# Deep Learning Convolutional Neural Networks Discriminate Adult ADHD From Healthy Individuals on the Basis of Event-Related Spectral EEG

**DOI:** 10.3389/fnins.2020.00251

**Published:** 2020-04-09

**Authors:** Laura Dubreuil-Vall, Giulio Ruffini, Joan A. Camprodon

**Affiliations:** ^1^Laboratory for Neuropsychiatry and Neuromodulation, Department of Psychiatry, Massachusetts General Hospital, Harvard Medical School, Boston, MA, United States; ^2^Department of Psychiatry and Clinical Psychobiology, Universitat de Barcelona, Barcelona, Spain; ^3^Neuroelectrics Corporation, Cambridge, MA, United States

**Keywords:** deep learning, EEG, ERP, ADHD, neuroimaging, brain disease diagnosis

## Abstract

Attention deficit hyperactivity disorder (ADHD) is a heterogeneous neurodevelopmental disorder that affects 5% of the pediatric and adult population worldwide. The diagnosis remains essentially clinical, based on history and exam, with no available biomarkers. In this paper, we describe a convolutional neural network (CNN) with a four-layer architecture combining filtering and pooling, which we train using stacked multi-channel EEG time-frequency decompositions (spectrograms) of electroencephalography data (EEG), particularly of event-related potentials (ERP) from ADHD patients (*n* = 20) and healthy controls (*n* = 20) collected during the Flanker Task, with 2800 samples for each group. We treat the data as in audio or image classification approaches, where deep networks have proven successful by exploiting invariances and compositional features in the data. The model reaches a classification accuracy of 88% ± 1.12%, outperforming the Recurrent Neural Network and the Shallow Neural Network used for comparison, and with the key advantage, compared with other machine learning approaches, of avoiding the need for manual selection of EEG spectral or channel features. The event-related spectrograms also provide greater accuracy compared to resting state EEG spectrograms. Finally, through the use of feature visualization techniques such as *DeepDream*, we show that the main features exciting the CNN nodes are a decreased power in the alpha band and an increased power in the delta-theta band around 100 ms for ADHD patients compared to healthy controls, suggestive of attentional and inhibition deficits, which have been previously suggested as pathophyisiological signatures of ADHD. While confirmation with larger clinical samples is necessary, these results suggest that deep networks may provide a useful tool for the analysis of EEG dynamics even from relatively small datasets, highlighting the potential of this methodology to develop biomarkers of practical clinical utility.

## Introduction

Attention-deficit hyperactivity disorder (ADHD) is a neurodevelopmental disorder characterized by deficits in attention, impulsivity (motor and non-motor) and executive dysfunction. It is associated with high morbidity and disability (Biederman et al., 2006; Shamay-Tsoory and Aharon-Peretz, 2007), and affects up to 5% of adults worldwide (Kessler et al., 2006; Fayyad et al., 2007; Polanczyk et al., 2007). The diagnosis of ADHD remains essentially clinical, based on history and exam. It can be supported by neuropsychological assessments, but given the heterogeneous cognitive profiles in patients with ADHD, these provide a supportive, not fully diagnostic, function. Significantly, there are many different conditions that present with disordered attention, impulsivity and dysexecutive syndromes, and the range of normal cognitive profiles with variable strengths and weaknesses in these domains is wide, often complicating the differential diagnosis. Hence, a biomarker to reduce the inherent uncertainty of clinical diagnosis would be of great value.

Electroencephalographic (EEG) signals contain rich information associated with functional dynamics in the brain. The use of EEG in ADHD began more than 75 years ago with Jasper et al. (1938) reporting an increase in the EEG power of low frequencies in fronto-central areas. Since then, human electrophysiological studies using EEG spectral analyses and event-related potentials (ERPs) have established relevant signatures of executive dysfunction in ADHD (Lenartowicz and Loo, 2014). In contrast to spontaneous EEG, ERPs reflect changes in the electrical activity of the brain that are time-locked to the occurrence of a specific event, that is, a response to a discrete external stimulus or an internal mental process (Fabiani et al., 2007). ERPs also provide non-invasive neurophysiological measurements with high temporal resolution, allowing to assess dysfunctional brain dynamics, including cognitive processes that may not be apparent at the behavioral level (Woodman, 2010; Sanei and Chambers, 2013). Indeed, ERPs are commonly used clinically in neurophysiological diagnostic units to support the assessment of neuropsychiatric disorders [e.g., multiple sclerosis (Pokryszko-Dragan et al., 2016)] and sensory disorders [e.g., screening of neonates for hearing impairments (Paulraj et al., 2015)].

Artificial neural networks have recently become a promising application of artificial intelligence (AI) in healthcare (Durstewitz et al., 2019). Machine learning, a subtype of AI, and deep learning, a specialized sub-field of machine learning, have been increasingly used in clinical research with promising results. Machine learning can be described as the practice of using algorithms to train a system by using large amounts of data, with the goal of giving it the ability to learn how to perform a specific task, and then make an accurate classification or prediction. Deep learning is a subset of machine learning algorithms that break down the tasks in smaller units (neural networks, NNs) often providing higher levels of accuracy.

Neural networks are characterized by their network architecture, defined by the anatomical layout of its connected processing units, the artificial “neurons,” according to a loss or optimization function that specifies the overall goal of the learning process. Connections are “trained,” or taught how to do the desired task, by using a training algorithm that iteratively changes parameters of the neural network such that the target function is ultimately optimized based on the inputs the neural network receives. There are different types of neural networks with different designs and architectures derived from different principles, or conceived for different purposes. The most basic ones are the feed-forward neural networks (FNNs), in which activity is propagated unidirectionally layer-by-layer from the input up to the output stage, with no feedback connections within or between layers. We have previously used a specific type of FFNs (feed-forward autoencoders) for the analysis of EEG data with promising outcomes (Kroupi et al., 2017). Recurrent Neural Networks (RNN) are another type of neural networks that, unlike FFNs, are based on architectures with feedback (“recurrent”) connections within or between layers. In related work, we used Echo State Networks (ESNs), a particular type of RNN, to classify Parkinson patients from HC using EEG time-frequency decompositions (Ruffini et al., 2016) with successful results. The main limitation of RNNs is, however, their computational cost (Goodfellow et al., 2016). In addition, one of the main critics to deep neural networks is their “black-box” nature, i.e., the difficulty in tracing a prediction back to which features are important and understanding how the network reached the final output, which will be later addressed in this study.

Previous studies have successfully classified ADHD patients from HC using machine learning techniques with accuracies of more than 90% (Ahmadlou and Adeli, 2010; Mueller et al., 2010; Sadatnezhad et al., 2011; Abibullaev and An, 2012; Nazhvani et al., 2013; Tenev et al., 2014; Jahanshahloo et al., 2017), but the selection of disease-characterizing features from EEG was done manually after an extensive search in the frequency or time domain. However, EEG signals exhibit non-linear dynamics (chaotic signals that do not behave linearly and cannot be represented as combination of basic sub-signals) and non-stationarity across temporal scales (signals with a mean and variance that do not stay constant over time) that cannot be studied properly using classical machine learning approaches. There is a need for tools capable of capturing the rich spatiotemporal hierarchical structures hidden in these signals. In a previous study (Ruffini et al., 2018), we trained a machine learning system with pre-defined complexity metrics of time-frequency decompositions of EEG data that showed statistically significant differences between REM Sleep Behavior Disorder (RBD) patients and HC, indicating that such metrics may be useful for classification or scoring. While this approach is useful in several domains, it would be advantageous to use methods where the relevant features are found directly by the algorithms instead of pre-defining them manually, which usually requires a costly post-processing of the EEG signals and an extensive time-consuming feature engineering process.

With the goal of building a discrimination system that can classify ADHD patients from HCs, here we explore a deep learning approach inspired by recent successes in image classification using Convolutional Neural Networks (CNNs), a particular type of NN designed to exploit compositional and translationally invariant features in the data that are present in EEG, i.e., features that are recognizable even if their appearance varies in some way (Goodfellow et al., 2016). These networks were originally developed to deal with image data (2D arrays) from different channels or audio data (Oord et al., 2013), and more recently, EEG data (Tsinalis et al., 2016; Vilamala et al., 2017). Similarly, here we train a CNN with multi-channel two-dimensional time-frequency maps (spectrograms), representing EEG spectral dynamics as images with the equivalent image depth provided by multiple EEG channels. These networks treat EEG-channel data as an audio file, and our approach mimics similar uses of deep networks in that domain. Specifically, we use a similar strategy as the one presented by Ruffini et al. (2019), but instead of using spontaneous EEG spectrograms, we use ERP spectrograms (also called Event-Related Spectral perturbation, ERSP) recorded during a Flanker-Eriksen Task (EFT), a well-established experimental task to assess sustained attention, conflict monitoring and response inhibition. Our assumption is that relevant qualities of ERP data are contained in compositional features embedded in this time-frequency representation. Particularly, we expect that CNNs may be able to efficiently learn to identify features in the time-frequency domain associated to event-related bursting across frequency bands that may help separate classes, similar to what is known as “bump analysis” (Dauwels et al., 2010). For comparison purposes, we also trained a RNN based on Long Short-Term Memory (LSTM) networks, which can learn long sequences of data but require higher computational demands, and a Shallow Neural Network (SNN), a more basic type of network with only one layer. We also compared the performance of the ERSP data with a dataset of spontaneous EEG data recorded while the participants were at resting state. Lastly, we propose the utilization of deep learning visualization techniques for the mechanistic interpretation of results, particularly the method popularly known as DeepDream (Alexander et al., 2015). This is important to identify pathophysiological features driving the translational and clinical value of the application, and for the optimized further development and acceptance of such techniques in the clinical domain, where black-box approaches have been extensively criticized.

## Materials and Methods

**FIGURE 1:**
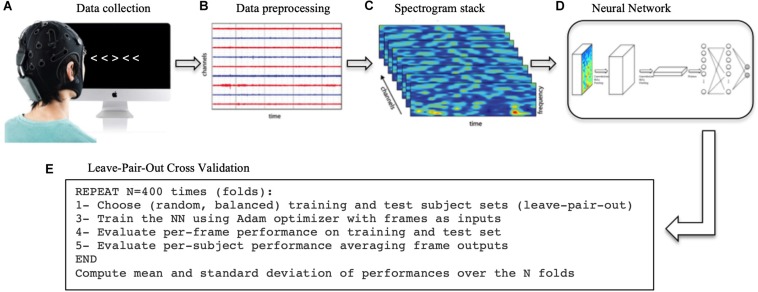
Methodological process. **(A)** First, the EEG data was collected while the subjects performed the Flanker task. **(B)** The data was then processed, filtered and cut into in epochs of 1 s time-locked at each trial to extract ERPs. **(C)** The Wavelet transform was applied to each single trial to extract the spectrograms. **(D)** The spectrogram stacks were input into the CNN (and RNN/SNN for comparison). **(E)** The network was trained 400 times (folds) using a different training/test sets each time. The network final performance is the average of the 400-folds performances.

A general overview of the methodological process is depicted in Figure 1.

### Participants

The dataset consisted of EEG data from a total of 40 participants including 20 healthy adults (10 males, 10 females) and 20 ADHD adult patients (10 males, 10 females) recorded while they were performing the EFT (Table 1). The inclusion criteria for ADHD patients consisted of a diagnosis of ADHD made by a board-certified clinician according to the Diagnostic and Statistical Manual of Mental Disorders, Fifth Edition (DSM-5) (American-Psychiatric-Association, 2013). Symptom profiles and severity were assessed with the Adult ADHD Self-Report Scale (ASRS-v1.1) (Kessler et al., 2005). Patients were either off stimulant medications or, if undergoing treatment with stimulants, were asked to discontinue 2 days prior to the experiment, under a physician-guided protocol, and allowed to resume afterward. Psychiatric comorbidities were allowed as long as ADHD was the primary diagnosis. Psychosis, bipolar disorder, substance use disorder and neurological conditions were exclusion criteria. Healthy participants were included if they did not have any psychiatric or neurologic condition and were not taking any psychoactive medications. All participants gave informed and written consent for participation. The study was approved by the Partners HealthCare System’s Institutional Review Board and all experiments were performed in accordance with relevant guidelines and regulations at Massachusetts General Hospital.

**TABLE 1:** Participant characteristics.

	ADHD (*n* = 20)	HC (*n* = 20)	Significance
Demographic	Mean (SD)*	Mean (SD)*	*p*-Value (*T*-test)
Age	43.85 (14.78)	29.90 (10.77)	0.0006
Females	10(50%)	10(50%)	0.5
**Baseline scores**			
ASRS	62.6 (9.17)	36.47 (11.33)	<0.0001
**Current medications – N (%)**			
No medication	11(55%)		
Adderall	2(15%)		
Vyvanse	2(10%)		
Concerta	1(5%)		
Verapamil	1(5%)		
Aspirin	1(5%)		
Levothyroxine	1(5%)		
Modafinil	1(5%)		

SD: Standard Deviation. (*) All figures are “mean (Standard Deviation)” unless otherwise specified.

### Experimental Task: Eriksen-Flanker Task (EFT)

**FIGURE 2:**
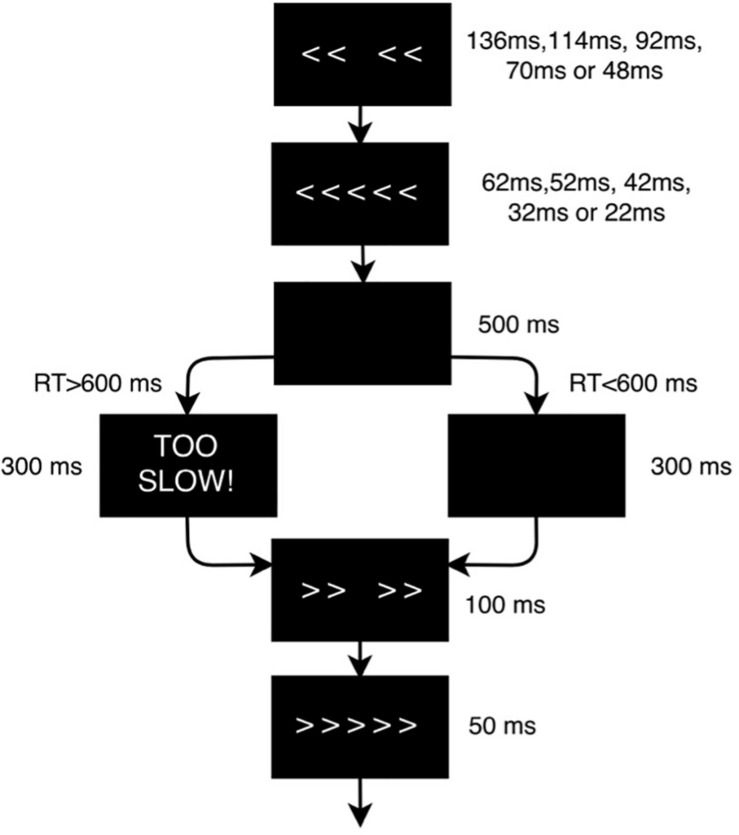
Flanker task design scheme. The flanker arrows were first presented alone for duration of 136, 114, 92, 70, or 48 ms depending on the baseline performance of each subject, and were then joined by the target arrow for 62, 52, 42, 32, or 22 ms, respectively (the duration of the stimuli was adjusted to the psychometric spot in which each subject reached a performance of 80–85%). Stimulus presentation was followed by a black screen for 500 ms. The time-window for participants’ response was 600 ms after target onset. If the participant did not respond within the response window, a screen reading ‘TOO SLOW!’ was presented for 300 ms. Participants were told that if they saw this screen, they should speed up. If a response was made before the deadline, the “TOO SLOW!” screen was omitted and the black screen remained on screen for the 300 ms interval. Finally, each trial ended with presentation of the fixation cross for an additional randomly chosen duration (200, 300 or 400 ms) in order to avoid any habituation or expectation by the subject. Thus, trial duration varied between 1070 and 1400 ms.

Each participant underwent three identical experimental sessions separated by 1–2 weeks in which they performed the EFT (Figure 2) while EEG data was recorded. The EFT is a classic behavioral paradigm in which subjects must attend and respond to the direction of a central arrow that is surrounded (“flanked”) by distracting stimuli. The flanking arrows can either have the same (congruent trials) or opposing (incongruent trials) orientation as the central one. Participants are instructed to press the left or right arrow buttons in a keyboard following the direction of the central arrow, ignoring the flankers. In this study there were a total of 140 trials in each session, and each subject had a different, fully random sequence of congruent and incongruent trials, with 2 congruent trials for each incongruent trial, in order to build a tendency toward the prepotent congruent responses and thus increase the difficulty of conflict detection in incongruent trials. Only incongruent trials were used for classification purposes, as they are the ones that most elicit the conflict-related ERP components that characterize the executive function subtasks of selective attention, inhibition and cognitive control (Kopp et al., 1996), primarily impaired in ADHD.

In order to help interpret the results of the extracted features, performance (percentage of correct/incorrect responses) and reaction time were statistically analyzed with R software (R Core Team, 2017). Reaction time of single trials was modeled with a Generalized Linear Model with Mixed Effects (GLMM) with a Gamma distribution, with Group as a fixed factor (ADHD/HC) and Subject ID as a random intercept. Accuracy was also modeled using a generalized logistic regression model with mixed effects and a binomial distribution. Changes were considered significant when *p* < 0.05.

### EEG Data Acquisition and Preprocessing

EEG was recorded with the Starstim system (Neuroelectrics, Cambridge, MA, United States) from 7 positions covering the primary hubs of the fronto-parietal executive control network (Fp1, Fp2, F3, Fz, F4, P3, and P4) with 3.14 cm^2^ Ag/AgCl electrodes and digitalized with 24-bit resolution at a sampling frequency of 500 samples/second. EEG data was referenced to the right mastoid. Independent component analysis (ICA) was utilized to identify and remove activity associated with blinks, eye movements, and other artifacts. Data was filtered from 1 to 20 Hz to remove non-neural physiological activity (skin/sweat potentials) and noise from electrical outlets. Trials were epoched within a time frame of 200 ms before and 800 ms after the stimulus onset. The mean of the pre-stimulus baseline [−200,0] ms was then subtracted from the entire ERP waveform for each epoch to eliminate any voltage offset.

To create the ERP spectrograms (or ERSP), the Wavelet transform was applied to each singe trial as implemented in EEGlab’s *newtimef* function, with 1 wavelet cycle at the lowest frequency to 10 cycles at the highest, leading to 22 frequency bins logarithmically spaced in the [3, 20] Hz range and 20 linear time bins in the [0, 800] ms range, where 0 represents the onset of the target stimuli in incongruent trials. The input data frames (or trials) were thus multidimensional arrays of the form [22 Frequency bins] × [20 Time bins] × [7 channels], with 140 data frames per subject (2800 data frames for each group). For comparison purposes, we also processed a dataset of spontaneous EEG data recorded while the same subjects and ADHD patients were resting with eyes closed (no cognitive task performed). For comparison purposes, the steps to preprocess the spontaneous EEG spectrograms were exactly the same as the ERP spectrograms, and the number of spectrograms was the same in both datasets (2800 per group). The only difference between both datasets is the fact the ERP spectrograms were time-locked to a stimulus that elicits the primary executive functions impaired in ADHD, while the spontaneous EEG spectrograms were just recorded while the subject was in resting state.

### Neural Network Implementation

#### Architecture

**FIGURE 3:**
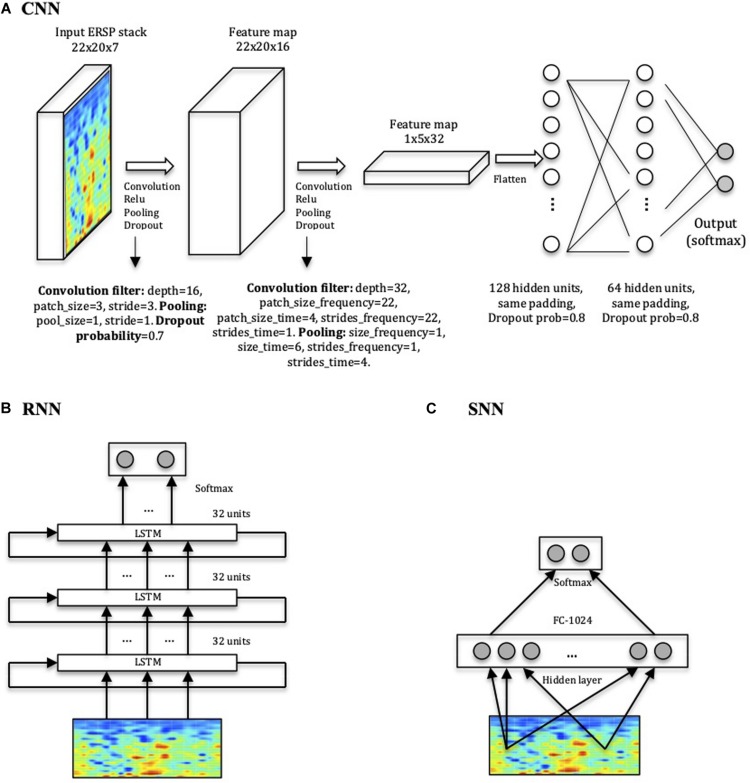
Network architectures. **(A)** CNN model displaying input, convolution with pooling layers, and hidden-unit layers. The first two layers perform the convolution, the Rectified Linear Units (ReLU) function and the pooling processes for feature extraction. The last two layers with 128 and 64 hidden nodes perform the class classification in HC or ADHD. **(B)** RNN consisting of three stacked layers of LSTM cells, where each cell uses as input the outputs of the previous one. Each cell used 32 hidden units, and dropout was used to regularize it. **(C)** SNN architecture used for comparison with one layer of 1024 units.

The CNN, implemented in Tensorflow (Abadi et al., 2016), is a relatively simple four-layer convolutional network (Figure 3A). The total number of parameters of the network is 75106. The patch size of the convolutional filter, the pooling and dropout parameters and the number of hidden units in the linear layers are indicated in Figure 3. We compared the CNN’s performance with a Shallow Neural Network (SNN) (Figure 3C), a more basic neural network with a hidden layer, and with a RNN consisting of stacked LSTM (Hochreiter and Schmidhuber, 1997; Goodfellow et al., 2016), a type of RNN capable of using information about events in the past (memory) to inform predictions in the future (Figure 3B). Model performances were statistically compared using a *t*-test, and were considered significant when *p* < 0.05.

#### Training

All weights were initialized from a zero-centered Normal distribution with standard deviation of 0.1. The model was trained using the Adam optimizer, an extension of stochastic gradient descent (Kingma and Ba, 2015), with a batch size of 32, number of training epochs of 600, a learning rate of 0.001 and momentum terms (β_1_ and β_2_) set at the default value of 0.9. These parameters were already used in our previous study (Ruffini et al., 2019) and were shown to provide a stable training process. The network was trained using a cross-entropy loss function to classify frames (not subjects), but it was evaluated on subjects by averaging subject frame scores and choosing the maximal probability class, i.e., using a 50% threshold. While this is somewhat an arbitrary decision threshold, it is natural to set it at 50% by default in this type of frameworks, where we work with a binary decision problem with balanced classes in training and test, and a softmax (probability-like) output.

#### Regularization

In order to avoid overfitting the data (i.e., overtraining the system to the extent that it negatively impacts the performance of the model on new data), we used the “Dropout” method, a regularization technique in which randomly selected neurons are ignored during training (Srivastava et al., 2014). The number of training epochs was also limited to the point after which more iterations did not improve training significantly and may lead to overfitting, a method known as “early stopping” (Prechelt, 1998).

### Performance Assessment

In order to assess the performance of the different architectures, the networks were trained 400 times (folds), holding one subject from each group out from the training set at a time, and measuring the performance using the held out pair as a test set. The final performance was the average of the 400-fold performances. Both the training and test sets were balanced in terms of subjects and frames per class. The number of folds was set to 400, corresponding to all possible combinations of pairing 1 HC with 1 ADHD (20 × 20). This method is known as leave-pair out cross validation (LPOCV) (Airola et al., 2009), a method for model selection and performance assessment of deep learning algorithms for small datasets. The advantage of the LPOCV approach is that the interactions of the various train-test partitions replace the need for a validation set, which may not be feasible to have in small datasets like this one.

The performance metrics assessed for each architecture were accuracy (ACC) and area under the curve (AUC) using the Wilcoxon-Mann-Whitney statistic (Airola et al., 2009). The ACC is defined as percentage of participants correctly classified, while the AUC of the corresponding receiver operating characteristic (ROC) curve is equal to the probability that a classifier will rank a randomly chosen positive instance higher than a randomly chosen negative one.

To account for the significant differences in age between the ADHD and HC groups, which could become a confounding factor, different approaches exist. Permutation tests is one of them, in which one randomly assigns a label to each data frame, trains the model and cross-validates it to compute overall classification accuracy. In our case, we should perform this process for every possible permutation (i.e., 400 permutations × 400 cross-validation folds). However, training a deep learning model 160,000 times is impractical and very time-consuming, and therefore we opted for the Inverse Probability Weighting (IPW) method (Linn et al., 2016), which assigns different weights to the subjects in the training process according to the inverse of their propensity score (Austin, 2011), which is defined as the probability of having ADHD given their age. In our specific dataset, the ages of the ADHD group are older than the healthy control group, which could become a potential confounding factor. To account for this, the IPW method will assign greater weights to those “rare” subjects that have ADHD despite being younger, and those subjects who do not have ADHD despite being older. Ultimately, this allows for the system to give more importance to those subjects that are less affected by age as a cofounding factor. After applying the IPW method, the performance of all architectures was the same as when no IPW adjustment was applied, thus ruling out the effect of age as a confounding factor.

### Feature Visualization

Once the network was trained, it was used to find out what type of inputs optimally excite the output nodes using a method popularly known as DeepDream (Alexander et al., 2015), which refers to the generation of synthetic images that produce desired activations in a trained deep network by exaggerating small features within them. The algorithm maximizes a particular class score using gradient descent, starting from a null or random noise image. In particular, we computed the DeepDream spectrograms averaged over 400-folds by maximizing the output logits after 30 iterations in steps of 1, initializing with different random images (seeds).

## Results

**FIGURE 4:**
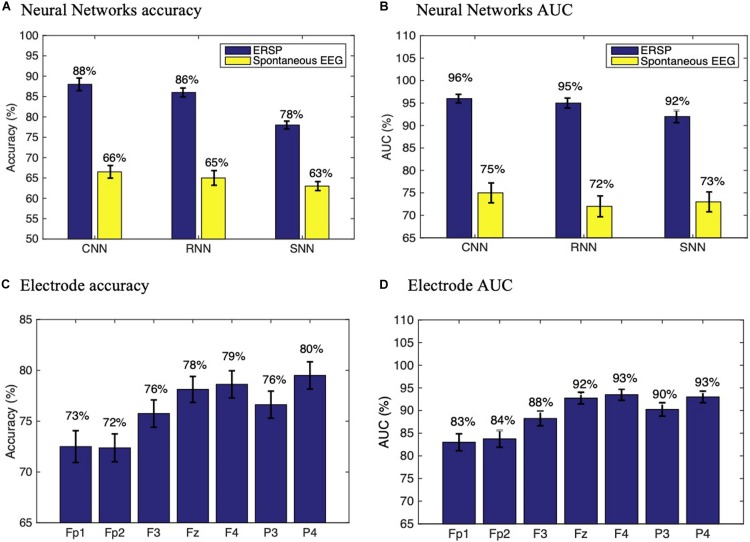
Performance assessment. Neural networks subject accuracy **(A)** and AUC **(B)** with ERSP and spontaneous EEG data. Subject accuracy **(C)** and AUC **(D)** for each electrode in a CNN trained with ERSP data from single channels. Experiments were repeated *n* = 10 times to ensure replicability.

The results from classification using different methods and datasets are detailed in Figures 4A,B, showing that the CNN trained with ERSPs reached a subject accuracy of 88 ± 1.12% (AUC = 96 ± 0.74%), significantly outperforming the RNN (Accuracy = 86 ± 1.17%, AUC = 95 ± 1.11%, *t* = −34, *p* < 0.0001) and the SNN (Accuracy = 78 ± 1.3%, AUC = 92 ± 1.35%, *t* = −132, *p* < 0.0001). In comparison with spontaneous EEG spectrograms, ERSPs provided significantly better performance for all architectures. To assess the performance of each individual channel, we also trained the CNN with ERSP data from single channels and found that frontal (F3, Fz and F4) and parietal electrodes (P3, P4) provide the best performance compared to frontopolar (Fp1, Fp2) electrodes (Figures 4C,D).

**FIGURE 5:**
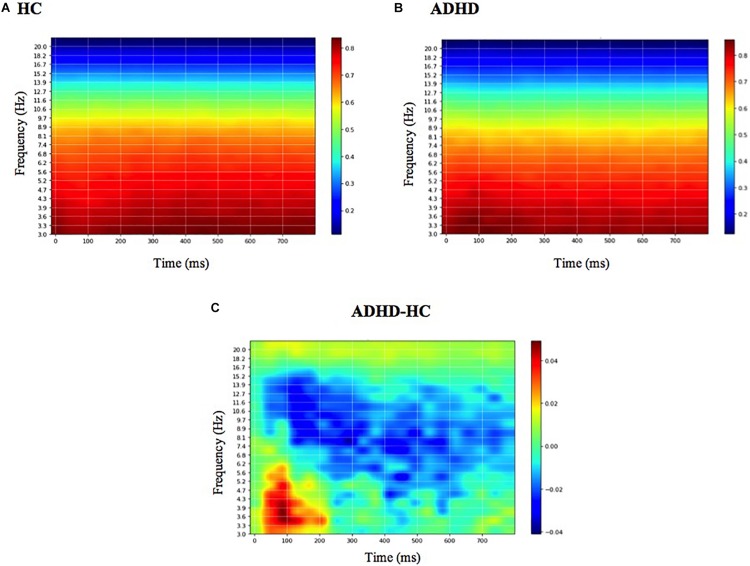
Mean DeepDream ERSP. Mean DeepDream ERSP averaged over channels and 400-folds for healthy controls **(A)**, ADHD patients **(B)** and their difference **(C)**. Color bar units = dB.

The mean DeepDream ERSP averaged over channels can be seen in Figure 5 (see Supplementary Table S1 for individual channels). The difference between groups reveals that the main feature that optimally excites the network nodes is an increased power for the ADHD group in the delta-theta band (3–7 Hz) around 100 ms and a decreased power in the alpha band (7–12 Hz) along the entire time course, with a residual decrease in theta and beta. Note that the patterns shown in the DeepDream ERSP are very similar to the patterns of the ERSP computed from the real data (Supplementary Table S1), thus showing that the network is actually learning real neurophysiologically identifiable differences between groups.

Behaviorally, the mean reaction time of the dataset was significantly slower for ADHD compared to HC (RT_ADHD_ = 368 ms, RT_HC_ = 321 ms, β = 46 ms, CI = [38,53] ms, *p* < 0.001), which can be expected with this type of population with attention deficits, but the mean percentage of correct responses for each group was not significantly different (Accuracy_ADHD_ = 62%, Accuracy_HC_ = 65%, β = 0.12, CI = [0.02, 0.26], *p* = 0.10).

## Discussion

In this study, we present a viable deep learning model for effective discrimination of patients with ADHD from healthy controls on the basis of ERSP during the Eriksen-Flanker task, providing a new tool for the analysis of EEG dynamics in ADHD and supporting the potential of deep learning strategies for biomarker development in neuropsychiatry. We deem this approach to be particularly interesting for various reasons. First, it largely mitigates the need for EEG feature selection (spectral bands, time ranges, specific ERP components, amplitudes, latencies, channels). Second, results with ERSPs seem to confer an advantage over spontaneous EEG spectrograms (e.g., subject accuracy with CNN was 88 ± 1.12% for ERSP vs. 66 ± 1.13% for spontaneous resting-state data). Third, the performance of the proposed CNN system significantly outperforms the RNN and the SNN used for comparison. Finally, through the use of feature visualization, we identify neurophysiologically interpretable features that can be extracted from the model, providing further validation and evidence that the network performance is not driven by noise or artifact signals in the data and providing a mechanistic model with added value to understand pathophysiology.

The higher accuracy provided by CNN and RNN compared to SNN proves that the complex deep approaches with more layers and units provide better performance than more shallow networks. The fact that CNN significantly outperforms RNN shows, however, that the higher computational demands of RNN do not provide better performance than the CNN approaches, thus proving CNN as a more efficient method than RNN. One possible reason why the RNN did not outperform the CNN may be because the temporal information about the way the EEG signals evolve over time (exploited by the RNN) is not as well represented in spectrograms as is the spatial information used by the CNN. In order to better leverage the long-short term memory capabilities of LSTMs and potentially improve the model performance, future work should include the combination of LSTMs and CNNs, for example by placing an LSTM before the input of the CNN.

The fact that ERSP data provide better performance than spontaneous EEG data with all architectures also shows that event-related data from a high yield task that probes critical executive functions impaired in ADHD is a better predictor than spontaneous EEG data recorded while the participants are at resting state. However, although the same preprocessing pipeline was applied to both datasets for comparison purposes, we acknowledge there may be a potential bias, as the optimal preprocessing for spontaneous EEG spectrograms may not be the same as for the ERP spectrograms. For example, similar studies using traditional machine learning techniques with resting state EEG power features have reached accuracies of 72% (Tenev et al., 2014), which suggests that our performance could possibly be improved if the preprocessing pipeline were optimized.

Finally, through the use of feature visualization we show that the main spectral features identified by the CNN nodes to classify ADHD patients from healthy controls are a decrease in faster frequencies (alpha band, 8–12 Hz) in frontal electrodes over the entire time course and an increase in slower frequencies (delta-theta band, 3–6 Hz) in frontal and parietal electrodes around 100 ms for ADHD patients compared to HC. There is evidence that alpha activity (or alpha Event-Related Synchronization, ERS) in conflict and inhibitory tasks is associated with the capacity to inhibit prepotent response, reflecting a top-down inhibitory control process (Klimesch et al., 2007). Thus, we interpret the decrease in alpha activity as a deficit in the inhibition of prepotent responses in ADHD, consistent with similar findings (Lenartowicz et al., 2018). On the other hand, the increased delta-theta activity is more specifically constrained to the 100 ms period, and is probably related to the increase in N100 amplitude in the time domain (Supplementary Table S1). N100 is a visual sensory evoked potential that is thought to reflect sensory analysis of simple stimulus features and whose amplitude is influenced by selective attention (Rugg et al., 1987): the greater the N100 amplitude, the greater attentional resources dedicated to that specific task. The increased delta-theta power in that latency suggests that ADHD patients needed to shift more attention to the task in order to accomplish similar levels of correct responses compared to the healthy group. Since it is well known that ADHD patients manifest specific alterations in the process of selective attention of visual task stimuli (Yordanova et al., 2006), we interpret this as a compensation strategy to offset these deficits by shifting more attention to the task (Broyd et al., 2005; Prox et al., 2007).

Note that the DeepDream spectrograms generated for Fp1 and Fp2 are substantially different and provide lower performance that the other positions (F3, F4, Fz, P3, P4), which may be explained by the lower signal quality of frontopolar positions due to blinks, muscle artifacts and sweat. The lower performance of F3 and P3 electrodes compared to F4 and P4 may also be related to the lower power scale in their DeepDream spectrogram, respectively (Supplementary Table S1). This may suggest the existence of inter-hemispheric differences in the features driving the discrimination between ADHD and HC.

Similar studies have explored the application of deep learning to EEG signals (Roy et al., 2019). For example, CNNs have been used for epilepsy prediction and monitoring (Liang et al., 2016), mental workload classification (Ma et al., 2017) and motor imagery classification (An et al., 2014; Bashivan et al., 2015; Tabar and Halici, 2017). Deep neural networks have also shown convincing results in classifying psychiatric disorders such as dementia (Vieira et al., 2017) and ADHD (Kuang et al., 2014; Kuang and He, 2014; Deshpande et al., 2015; Han et al., 2015; Hao et al., 2015; Zou et al., 2017), mostly with MRI data, which is more costly to obtain than EEG data. To our knowledge, this is the first study using a deep learning approach with EEG event-related spectral data to discriminate adult ADHD patients from HC with no prior selection of EEG features and to incorporate feature visualization techniques to provide further mechanistic evidence of the underlying pathophysiology driving the classification. This is particularly important, as it not only allows to develop clinical tools but also to delineate pathological signatures and disease mechanisms.

One of the limitations of this study is the relatively small size of the dataset (2800 × 2 samples) which, coupled with the large number of parameters of the network (75106), risks the consequent susceptibility of overfitting. Although other regularization methods (apart from early stopping and dropout) could mitigate this issue and further improvements could be achieved with bigger datasets, our aim in this study was to implement a proof of concept before gathering larger amounts of training data. Now that the system has been validated, future work should include bigger training datasets as well as further regularization techniques that more closely prevent overfitting. Another limitation is the age difference between the two groups. While the mean ages are well after the period of brain maturation when myelination and ADHD symptoms are still changing, and well before a geriatric threshold when other type of biological changes (including normal aging) may affect cognition, we addressed this possible confounder using Inverse Probability Weighting. The age difference was an artifact caused by the fact that the two cohorts were recruited prospectively for independent studies (though at the same time and with the same exact protocol and hardware) and then analyzed together retrospectively to address the proposed questions, hence the lack of appropriately age-matched controls. Future prospective validation studies should use larger cohorts and randomize age-matched controls. Finally, the fact that the classifier has a lower accuracy than AUC indicates that there may be a bias toward the ADHD class and therefore there may be other decision thresholds different than 50% that lead to better classification performance. In a real setting, the final decision threshold to be used will depend on the actual application scenario, and it will need to be adapted according to the balancing between the penalties for false positives and false negatives.

It is also worth mentioning that, although the current work considerably eliminates the need for manual extraction of features, it is still focused on classification during high yield incongruent trials of a specific task. While this requires *a priori* knowledge constrains, if validated with bigger datasets (and possibly higher definition EEG), it may be a helpful diagnostic and biomarker development strategy (i.e., choosing high yield events of a high yield disease-relevant task) with practical future procedural advantages (i.e., it would be easy to implement it in clinical settings with currently existing tools, such as tasks for neuropsychological assessments and standard EEG for electrophysiological diagnosis).

Our findings may have several implications from the clinical perspective by bringing new information to inform the clinician’s decisions. Although the networks in this study have been trained with a small dataset of 40 subjects, if validated with bigger datasets this approach could be used to support the diagnosis of ADHD on a single-patient basis. The fact that the current networks have been trained with low-resolution EEG datasets (7 channels) of short duration (3 min) would make it easy to implement them not only in an EEG clinical unit, but possibly by an outpatient clinician, eliminating the need to get longer or higher quality data with sophisticated and clinically unpractical EEG systems. However, even if these deep learning systems are properly validated in the future, clinicians should view their output as statistical predictions, not as a ground truth, and they should judge whether the prediction applies to that specific patient and decide if additional data or expertise is needed to inform that decision.

Future work should include the exploration of this approach with larger datasets as well as a more systematic study of network architecture and regularization schemes. This includes the use of deeper architectures, improved data augmentation methods, alternative data segmentation and normalization schemes. With regards to data preprocessing, we should consider improved spectral estimation using more advanced techniques such as state-space estimation and multitapering (Kim et al., 2018), and the use of cortical or scalp-mapped EEG data prior creation of spectrograms.

It is worth noting that previous studies have successfully classified ADHD patients from HC using classical machine learning techniques applied to EEG data, achieving even higher performances. For example, (Mueller et al., 2010) reached a sensitivity and specificity of 91% in predicting ADHD diagnosis in a sample of 150 adults (75 with ADHD), using a combination of five response-inhibition ERP features identified using ICA. In a smaller sample (*n* = 36) (Nazhvani et al., 2013) developed an algorithm that identified the combination of time points at which the ERP amplitude maximized the accuracy of group discrimination, reaching an accuracy of 94.6% in discriminating adults with ADHD from controls. Similarly, using a combination of spectral power and fractal features of EEG time series, (Sadatnezhad et al., 2011) reported diagnostic accuracy of 86.4%, with fractal features leading to the strongest discrimination. Another study (Ahmadlou and Adeli, 2010) found maximal accuracy of 95.6% based on the combination of theta band synchronization at occipital and frontal electrodes, and delta band synchronization at electrode T5 and frontal electrodes. (Abibullaev and An, 2012) obtained a maximal accuracy of 97%, using relative theta measures recorded from nine frontal scalp electrodes. However, all these models required the manual selection of disease-characterizing features from EEG after an extensive search in the frequency or time domain. Thus, we did not compare the performance of the current model directly with classical machine learning models as our goal was not to outperform those models, but rather to validate the idea that deep learning approaches can provide value for the analysis of time-frequency representations of EEG, and particularly ERSP data, for the effective discrimination of ADHD.

Finally, we note that we make no attempt to fully-optimize our architecture in this study, nor to compare or improve the performance over other machine learning systems. In particular, no fine-tuning of hyper-parameters has been carried out using a validation set approach, a task we reserve for future work with larger datasets.

## Data Availability Statement

The data that support the findings of this study are available from the senior author, JC, upon reasonable request.

## Ethics Statement

The studies involving human participants were reviewed and approved by the Partners Healthcare Institutional Review Board. The patients/participants provided their written informed consent to participate in this study.

## Author Contributions

LD-V contributed with the processing of the data, the implementation of the deep learning systems, and the writing of the manuscript. GR contributed with the conception and design of the deep learning systems and the revision of the manuscript. JC contributed with the conception and design of the study, the supervision of data acquisition and the findings, and the critical revision of the manuscript. All authors gave the approval to the final version of the manuscript to be published.

## Conflict of Interest

LD-V is an employee at Neuroelectrics and a Ph.D. student in the Camprodon Lab. GR is a co-founder of Neuroelectrics, a company that manufactures the EEG device used in this study. JC is a member of the scientific advisory board for Apex Neuroscience Inc.

## References

[B1] Abadi M. Barham P. Chen J. Chen Z. Davis A. Dean J. (2016). “TensorFlow: a system for large-scale machine learning,” in *Proceedings of the 12th USENIX Conference on Operating Systems Design and Implementation* (Savannah, GA: USENIX Association).

[B2] Abibullaev B. An J. (2012). Decision support algorithm for diagnosis of ADHD using electroencephalograms. *J. Med. Syst.* 36 2675–2688. 10.1007/s10916-011-9742-x 21671069

[B3] Ahmadlou M. Adeli H. (2010). Wavelet-synchronization methodology: a new approach for EEG-based diagnosis of ADHD. *Clin. EEG Neurosci.* 41 1–10. 10.1177/155005941004100103 20307009

[B4] Airola A. Pahikkala T. Waegeman W. Baets B. D. Salakoski T. (2009). “A comparison of AUC estimators in small-sample studies,” in *Proceedings of the third International Workshop on Machine Learning in Systems Biology*, Ljubljana, 3–13.

[B5] Alexander M. Christopher O. Mike T. (2015). *DeepDream - a Code Example for Visualizing Neural Networks. Google Research Blog.* Available online at: https://ai.googleblog.com/2015/07/deepdream-code-example-for-visualizing.html (accessed December, 2019).

[B6] American-Psychiatric-Association (2013). *The Diagnostic and Statistical Manual of Mental Disorders.* Washington, DC: APA.

[B7] An X. Kuang D. Guo X. Zhao Y. He L. (2014). “A deep learning method for classification of EEG data based on motor imagery,” in *Proceedings of the 10th International Conference ICIC Intelligent Computing in Bioinformatics*, Taiyuan, 203–210. 10.1007/978-3-319-09330-7_25

[B8] Austin P. C. (2011). An introduction to propensity score methods for reducing the effects of confounding in observational studies. *Multivariate Behav. Res.* 46 399–424. 10.1080/00273171.2011.568786 21818162 PMC3144483

[B9] Bashivan P. Rish I. Yeasin M. Codella N. (2015). Learning Representations from EEG with Deep Recurrent-Convolutional Neural Networks. *arXiv* [Preprint]. Available online at: https://arxiv.org/abs/1511.06448 (accessed July, 2019).

[B10] Biederman J. Faraone S. V. Spencer T. J. Mick E. Monuteaux M. C. Aleardi M. (2006). Functional impairments in adults with self-reports of diagnosed ADHD: a controlled study of 1001 adults in the community. *J. Clin. Psychiatry* 67 524–540. 10.4088/jcp.v67n0403 16669717

[B11] Broyd S. J. Johnstone S. J. Barry R. J. Clarke A. R. Mccarthy R. Selikowitz M. (2005). The effect of methylphenidate on response inhibition and the event-related potential of children with attention deficit/hyperactivity disorder. *Int. J. Psychophysiol.* 58 47–58. 10.1016/j.ijpsycho.2005.03.008 15925419

[B12] Dauwels J. Vialatte F. Musha T. Cichocki A. (2010). A comparative study of synchrony measures for the early diagnosis of Alzheimer’s disease based on EEG. *Neuroimage* 49 668–693. 10.1016/j.neuroimage.2009.06.056 19573607

[B13] Deshpande G. Wang P. Rangaprakash D. Wilamowski B. (2015). Fully connected cascade artificial neural network architecture for attention deficit hyperactivity disorder classification from functional magnetic resonance imaging data. *IEEE Trans. Cybern* 45, 2668–2679. 10.1109/TCYB.2014.2379621 25576588

[B14] Durstewitz D. Koppe G. Meyer-Lindenberg A. (2019). Deep neural networks in psychiatry. *Mol. Psychiatry* 24, 1583–1598. 30770893 10.1038/s41380-019-0365-9

[B15] Fabiani M. Gratton G. Federmeier K. D. (2007). “Event-related brain potentials: methods, theory, and applications,” in *Handbook of Psychophysiology*, 3rd Edn (New York, NY: Cambridge University Press), 85–119. 10.1017/cbo9780511546396.004

[B16] Fayyad J. De Graaf R. Kessler R. Alonso J. Angermeyer M. Demyttenaere K. (2007). Cross-national prevalence and correlates of adult attention-deficit hyperactivity disorder. *Br. J. Psychiatry* 190 402–409. 17470954 10.1192/bjp.bp.106.034389

[B17] Goodfellow I. Bengio Y. Courville A. (2016). *Deep Learning.* Cambridge, MA: MIT Press.

[B18] Han X. Zhong Y. He L. Yu P. S. Zhang L. (2015). The unsupervised hierarchical convolutional sparse auto-encoder for neuroimaging data classification. *Brain Informatics Health* 156–166. 10.1007/978-3-319-23344-4_16

[B19] Hao A. J. He B. L. Yin C. H. (2015). “Discrimination of ADHD children based on deep bayesian network,” in *Proceedings of the 2015 IET International Conference on Biomedical Image and Signal Processing (ICBISP 2015)*, Beijing, 1–6.

[B20] Hochreiter S. Schmidhuber J. (1997). Long short-term memory. *Neural Comput.* 9 1735–1780. 9377276 10.1162/neco.1997.9.8.1735

[B21] Jahanshahloo H. R. Shamsi M. Ghasemi E. Kouhi A. (2017). Automated and ERP-based diagnosis of attention-deficit hyperactivity disorder in children. *J. Med. Signals Sens.* 7 26–32. 28487830 PMC5394803

[B22] Jasper H. H. Solomon P. Bradley C. (1938). Electroencephalographic analyses of behavior problem children. *Am. J. Psychiatry* 95 641–658. 10.1176/ajp.95.3.641

[B23] Kessler R. C. Adler L. Ames M. Demler O. Faraone S. Hiripi E. (2005). The World Health Organization Adult ADHD Self-Report Scale (ASRS): a short screening scale for use in the general population. *Psychol. Med.* 35 245–256. 10.1017/s0033291704002892 15841682

[B24] Kessler R. C. Adler L. Barkley R. Biederman J. Conners C. K. Demler O. (2006). The prevalence and correlates of adult ADHD in the United States: results from the National Comorbidity Survey Replication. *Am. J. Psychiatry* 163 716–723. 10.1176/ajp.2006.163.4.716 16585449 PMC2859678

[B25] Kim S.-E. Behr M. K. Ba D. Brown E. N. (2018). State-space multitaper time-frequency analysis. *Proc. Natl. Acad. Sci. U.S.A.* 115E5. 10.1073/pnas.1702877115 29255032 PMC5776784

[B26] Kingma D. P. Ba J. (2015). *Adam: A Method for Stochastic Optimization.* Available online at: https://arxiv.org/pdf/1412.6980.pdf (accessed July, 2019).

[B27] Klimesch W. Sauseng P. Hanslmayr S. (2007). EEG alpha oscillations: the inhibition–timing hypothesis. *Brain Res. Rev.* 53 63–88. 10.1016/j.brainresrev.2006.06.003 16887192

[B28] Kopp B. Rist F. Mattler U. (1996). N200 in the Flanker task as a neurobehavioral tool for investigating executive control. *Psychophysiology* 33 282–294. 10.1111/j.1469-8986.1996.tb00425.x 8936397

[B29] Kroupi E. Soria-Frisch A. Castellano M. David I.-S. Montplaisir J. Gagnon J.-F. (2017). “Deep networks using auto-encoders for PD prodromal analysis,” in *Proceedings of the HBP Student Conference on Transdisciplinary Research Linking Neuroscience, Brain Medicine and Computer Science*, Vienna.

[B30] Kuang D. Guo X. An X. Zhao Y. He L. (2014). Discrimination of ADHD Based on fMRI Data with Deep Belief Network. *Intell. Comput. Bioinformatics* 225–232. 10.1007/978-3-319-09330-7_27

[B31] Kuang D. He L. (2014). “Classification on ADHD with deep learning,” in *Proceedings of the 2014 International Conference on Cloud Computing and Big Data*, Wuhan, 27–32.

[B32] Lenartowicz A. Loo S. K. (2014). Use of EEG to Diagnose ADHD. *Curr. Psychiatry Rep.* 16:498. 10.1007/s11920-014-0498-0 25234074 PMC4633088

[B33] Lenartowicz A. Mazaheri A. Jensen O. Loo S. K. (2018). Aberrant modulation of brain oscillatory activity and attentional impairment in attention-deficit/hyperactivity disorder. *Biol. Psychiatry Cogn. Neurosci. Neuroimaging* 3 19–29. 10.1016/j.bpsc.2017.09.009 29397074 PMC5801762

[B34] Liang J. Lu R. Zhang C. Wang F. (2016). “Predicting seizures from electroencephalography recordings: a knowledge transfer strategy,” in *Proceedings of the IEEE International Conference on Healthcare Informatics (ICHI)*, Piscataway, NJ.

[B35] Linn K. A. Gaonkar B. Doshi J. Davatzikos C. Shinohara R. T. (2016). Addressing confounding in predictive models with an application to neuroimaging. *Int. J. Biostat.* 12 31–44. 10.1515/ijb-2015-0030 26641972 PMC5154735

[B36] Ma T. Li H. Yang H. Lv X. Li P. Liu T. (2017). The extraction of motion-onset VEP BCI features based on deep learning and compressed sensing. *J. Neurosci. Methods* 275 80–92. 10.1016/j.jneumeth.2016.11.002 27845150

[B37] Mueller A. Candrian G. Kropotov J. D. Ponomarev V. A. Baschera G. M. (2010). Classification of ADHD patients on the basis of independent ERP components using a machine learning system. *Nonlinear Biomed. Phys.* 4:S1. 10.1186/1753-4631-4-S1-S1 20522259 PMC2880795

[B38] Nazhvani A. D. Boostani R. Afrasiabi S. Sadatnezhad K. (2013). Classification of ADHD and BMD patients using visual evoked potential. *Clin. Neurol. Neurosurg.* 115 2329–2335. 10.1016/j.clineuro.2013.08.009 24050849

[B39] Oord A. V. D. Dieleman S. Schrauwen B. (2013). “Deep content-based music recommendation,” in *Proceedings of the 26th International Conference on Neural Information Processing Systems*, Vol. 2 (Red Hook, NY: Curran Associates Inc).

[B40] Paulraj M. P. Subramaniam K. Yaccob S. B. Adom A. H. Hema C. R. (2015). Auditory evoked potential response and hearing loss: a review. *Open Biomed. Eng. J.* 9 17–24. 10.2174/1874120701509010017 25893012 PMC4391208

[B41] Pokryszko-Dragan A. Zagrajek M. Slotwinski K. Bilinska M. Gruszka E. Podemski R. (2016). Event-related potentials and cognitive performance in multiple sclerosis patients with fatigue. *Neurol. Sci.* 37 1545–1556. 10.1007/s10072-016-2622-x 27271940 PMC4992503

[B42] Polanczyk G. De Lima M. S. Horta B. L. Biederman J. Rohde L. A. (2007). The worldwide prevalence of ADHD: a systematic review and metaregression analysis. *Am. J. Psychiatry* 164 942–948. 10.1176/ajp.2007.164.6.942 17541055

[B43] Prechelt L. (1998). “Early Stopping - But When?,” in *Neural Networks: Tricks of the Trade*, eds Orr G. B. Müller K.-R. (Berlin: Springer), 55–69. 10.1007/3-540-49430-8_3

[B44] Prox V. Dietrich D. E. Zhang Y. Emrich H. M. Ohlmeier M. D. (2007). Attentional processing in adults with ADHD as reflected by event-related potentials. *Neurosci. Lett.* 419 236–241. 10.1016/j.neulet.2007.04.011 17466456

[B45] R Core Team (2017). *A Language and Environment for Statistical Computing.* Vienna: R Foundation for Statistical Computing.

[B46] Roy Y. Banville H. Albuquerque I. Gramfort A. Falk T. H. Faubert J. (2019). Deep learning-based electroencephalography analysis: a systematic review. *J. Neural. Eng.* 16:051001. 10.1088/1741-2552/ab260c 31151119

[B47] Ruffini G. Ibañez D. Castellano M. Dubreuil-Vall L. Soria-Frisch A. Postuma R. (2019). Deep learning with EEG spectrograms in rapid eye movement behavior disorder. *Front. Neurol.* 10:806. 10.3389/fneur.2019.00806 31417485 PMC6683849

[B48] Ruffini G. Ibañez D. Castellano M. Dunne S. Soria-Frisch A. (2016). “EEG-driven RNN classification for prognosis of neurodegeneration in at-risk patients,” in *Proceedings of the ICANN 2016*, Barcelona, 306–313. 10.1007/978-3-319-44778-0_36

[B49] Ruffini G. Ibanez D. Kroupi E. Gagnon J.-F. Montplaisir J. Postuma R. B. (2018). Algorithmic complexity of EEG for prognosis of neurodegeneration in idiopathic rapid eye movement behavior disorder (RBD). *bioRxiv* [Preptint]. 10.1007/s10439-018-02112-0 30167913

[B50] Rugg M. D. Milner A. D. Lines C. R. Phalp R. (1987). Modulation of visual event-related potentials by spatial and non-spatial visual selective attention. *Neuropsychologia* 25 85–96. 10.1016/0028-3932(87)90045-5 3574653

[B51] Sadatnezhad K. Boostani R. Ghanizadeh A. (2011). Classification of BMD and ADHD patients using their EEG signals. *Expert Syst. Appl.* 38 1956–1963. 10.1016/j.eswa.2010.07.128

[B52] Sanei S. Chambers J. A. (2013). *EEG Signal Processing.* Hoboken, NJ: John Wiley & Sons Ltd.

[B53] Shamay-Tsoory S. G. Aharon-Peretz J. (2007). Dissociable prefrontal networks for cognitive and affective theory of mind: a lesion study. *Neuropsychologia* 45 3054–3067. 10.1016/j.neuropsychologia.2007.05.021 17640690

[B54] Srivastava N. Hinton G. Krizhevsky A. Sutskever I. Salakhutdinov R. (2014). Dropout: a simple way to prevent neural networks from overfitting. *J. Mach. Learn. Res.* 15 1929–1958.

[B55] Tabar Y. R. Halici U. (2017). A novel deep learning approach for classification of EEG motor imagery signals. *J. Neural Eng.* 14:016003. 10.1088/1741-2560/14/1/016003 27900952

[B56] Tenev A. Markovska-Simoska S. Kocarev L. Pop-Jordanov J. Muller A. Candrian G. (2014). Machine learning approach for classification of ADHD adults. *Int. J. Psychophysiol.* 93 162–166. 10.1016/j.ijpsycho.2013.01.008 23361114

[B57] Tsinalis O. Matthews P. M. Guo Y. Zafeiriou S. (2016). Automatic Sleep Stage Scoring with Single-Channel EEG Using Convolutional Neural Networks. *arXiv* [Preprint]. Available online at: https://arxiv.org/abs/1610.01683 (acessed July, 2019).

[B58] Vieira S. Pinaya W. H. Mechelli A. (2017). Using deep learning to investigate the neuroimaging correlates of psychiatric and neurological disorders: methods and applications. *Neurosci. Biobehav. Rev.* 74 58–75. 10.1016/j.neubiorev.2017.01.002 28087243

[B59] Vilamala A. Madsen K. H. Hansen L. K. (2017). “Deep convolutional neural networks for interpretable analysis of EEG sleep stage scoring,” in *Proceedings of the 2017 International workshop on Machine Learning for Signal Processing*, Tokyo.

[B60] Woodman G. F. (2010). A brief introduction to the use of event-related potentials in studies of perception and attention. *Atten. Percept. Psychophys.* 72 2031–2046. 10.3758/APP.72.8.2031 21097848 PMC3816929

[B61] Yordanova J. Heinrich H. Kolev V. Rothenberger A. (2006). Increased event-related theta activity as a psychophysiological marker of comorbidity in children with tics and attention-deficit/hyperactivity disorders. *Neuroimage* 32 940–955. 10.1016/j.neuroimage.2006.03.056 16730196

[B62] Zou L. Zheng J. Miao C. Mckeown M. J. Wang Z. J. (2017). 3D CNN based automatic diagnosis of attention deficit hyperactivity disorder using functional and structural MRI. *IEEE Access* 5 23626–23636. 10.1109/access.2017.2762703

