# Supplementary figures and images for: Deep Learning Convolutional Neural Networks Discriminate Adult ADHD From Healthy Individuals on the Basis of Event-Related Spectral EEG
Source: Front Neurosci. 2020 Apr 9;14:251. doi: 10.3389/fnins.2020.00251 (PMC7160297; doi:10.3389/fnins.2020.00251)

## Supplemental material

**Table S1.** Topography of group differences in ERSP and ERP per channel.

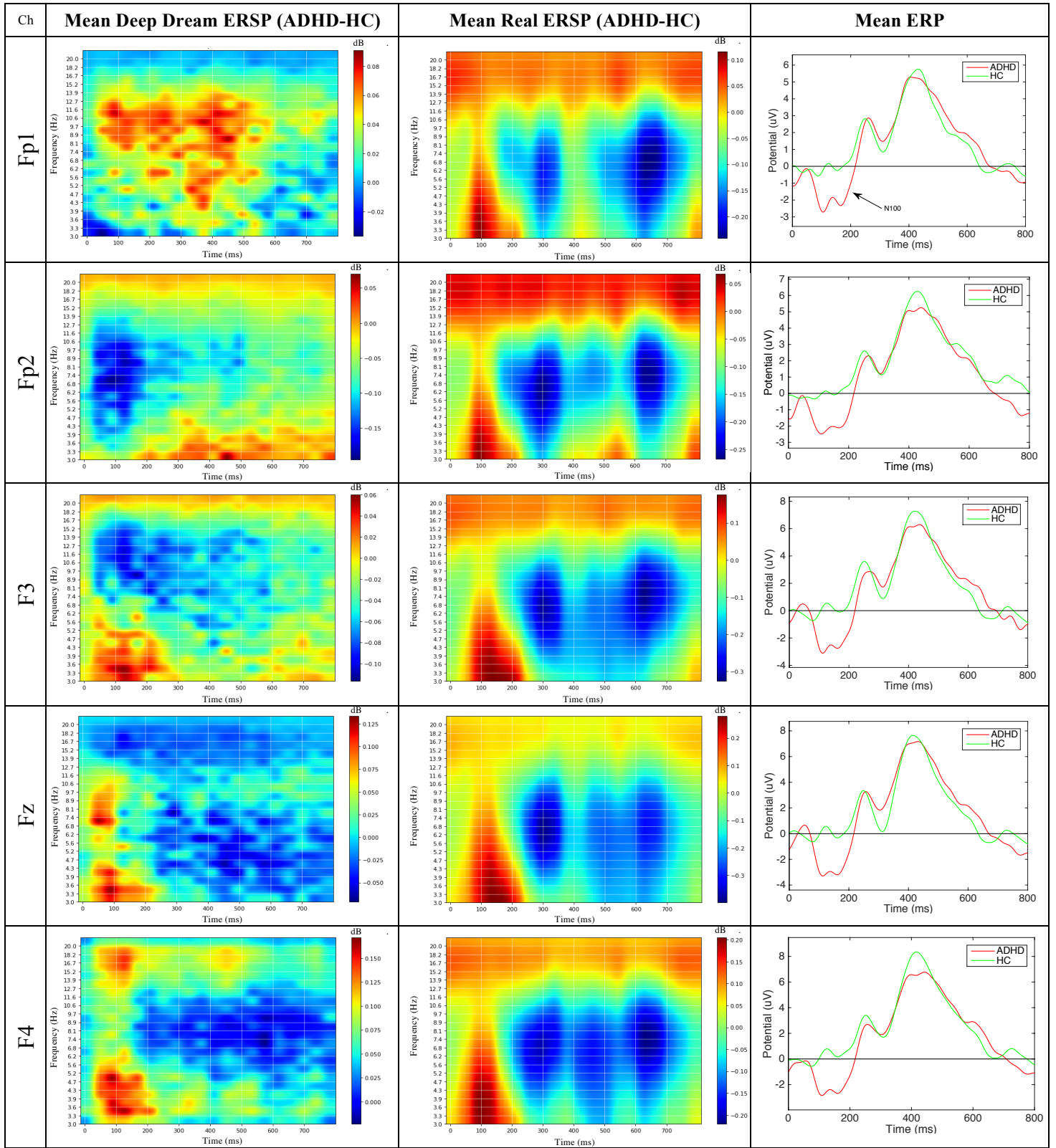

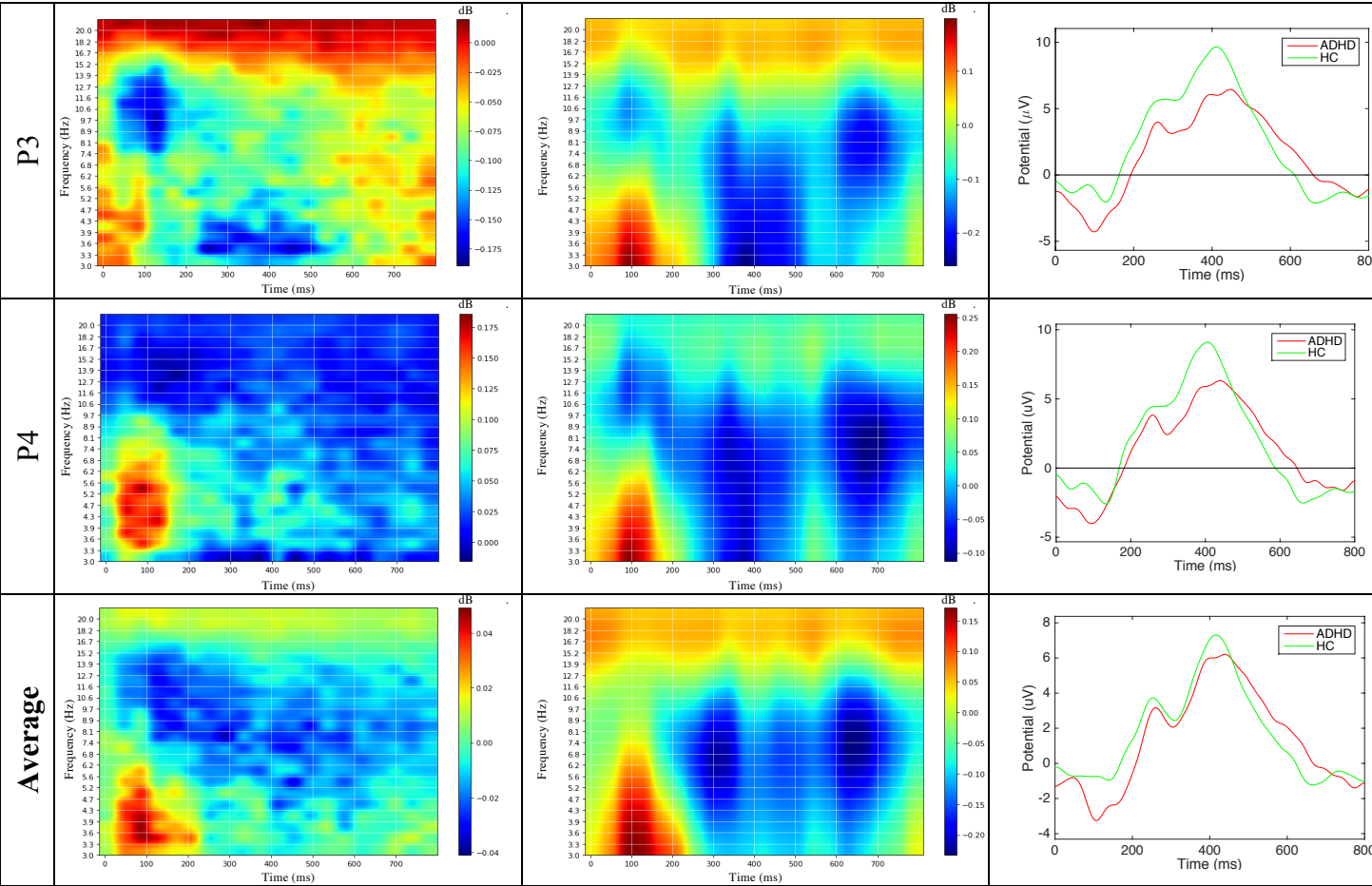

Supplement: Supplementary file 1 [file Data_Sheet_1.PDF]
